# Supplementary material for: Changes in the Infant Mortality Rate in Twin Towns of Brazil: An Ecological Study
Source: Children (Basel). 2022 Oct 30;9(11):1662. doi: 10.3390/children9111662 (PMC9688833; doi:10.3390/children9111662)
Supplement: Supplementary file 1 [file children-09-01662-s001.zip › children-1915106-supplementary.pdf]

Table **S1** – List of 33 Twin Cities, Federative Units and Bordering Border Municipalities.

CITIES	UF	BORDERING BORDER MUNICIPALITIES
**NORTH REGION**		
**1. ASSIS BRASIL**	AC	Sena Madureira, Bolpebra (Bolívia), Iñapari (Peru).
**2. BRASILÉIA**	AC	Sena Madureira (Bolívia)
**3. EPITACIOLÂNDIA**	AC	Cobija (Bolívia)
**4. SANTA ROSA DO PURUS**	AC	Peru
**5. OIAPOQUE**	AP	São Jorge do Oiapoque (Guiana Francesa)
**6. TABATINGA**	AM	Letícia (Colômbia)
**7. GUAJARÁ-MIRIM**	RO	Guayaramerín (Bolívia)
**8. PACARAIMA**	RR	Venezuela (Santa Elena de Uairén, Bolívar)
**9. BONFIM**	RR	Lethem (Guiana Inglesa)
**MIDWEST REGION**		
**10. CÁCERES**	MT	San Matias (Bolívia)
**11. BELA VISTA**	MS	Cerro Corá (Paraguai).
**12. CORUMBÁ**	MS	Puerto Suárez (Paraguai)
**13. MUNDO NOVO**	MS	Salto del Guairá (Paraguai)
**14. PARANHOS**	MS	Ypehú (Paraguai)
**15. PONTA PORÃ**	MS	Pedro Juan Caballero (Paraguai)
**16. CORONEL SAPUCAIA**	MS	Capitán Bado (Paraguai)
**17. PORTO MURTINHO**	MS	Porto Carmelo Peralta (Paraguai)
**SOUTH REGION**		
**18. BARRACÃO**	PR	Bernardo de Irigoyen (Missões, Argentina) e Dionísio Cerqueira (Brasil),
**19. FOZ DO IGUAÇU**	PR	Ciudad del Este (Brasil), Presidente Franco e Hernandarias (Paraguai) e Puerto Iguazú na Argentina.
**20. SANTO ANTÔNIO DO SUDOESTE**	PR	San Antonio (Misiones, Argentina)
**21. GUAÍRA**	PR	Salto del Guairá (Paraguai)
**22. DIONÍSIO CERQUEIRA**	SC	Bernardo de Irigoyen (Argentina)
**23. ACEGUÁ**	RS	Aceguá (Uruguai)
**24. BARRA DO QUARAÍ**	RS	Uruguaiana (Brasil), Bella Unión (Uruguai), Monte Caseros (Argentina)
**25. CHUÍ**	RS	Chuy (Uruguai)
**26. ITAQUI**	RS	La Cruz e Alvear (Argentina)
**27. JAGUARÃO**	RS	Rio Branco (Uruguai)
**28. PORTO XAVIER**	RS	San Javier (Argentina)
**29. QUARAÍ**	RS	Artigas (Uruguai)
**30. SANTANA DO LIVRAMENTO**	RS	Rivera (Uruguai)
**31. SÃO BORJA**	RS	Santo Tomé (Argentina)
**32. URUGUAIANA**	RS	Artigas (Uruguai) e Paso de los Libres e Yapeyú (Argentina)
**33. PORTO MAUÁ**	RS	Alba Posse (Argentina)

Table S2 Sociodemographic characteristics of the Brazilian Twin Cities, according to the HDI-M ranges, the Gini index and the coverage of the family health strategy, Brazil.

CITIES	STATE	NUMBER OF INHABITANTS		HDI-M	GINI INDEX			ESF COVERAGE (%)		
		2010	2019	2010	1991	2000	2010	2007	2010	2020
**NORTH REGION**										
1. ASSIS BRASIL	AC	6.072	7.417	0,588	0,457	0,578	0,622	100	100	100
2. BRASILÉIA	AC	21.398	26.278	0,614	0,571	0,614	0,594	100	100	100
3. EPITACIOLÂNDIA	AC	15.100	18.411	0,653	-	0,566	0,607	100	100	100
4. SANTA ROSA DO PURUS	AC	4.691	6.540	0,517	-	0,573	0,775	96,29	79,16	100
5. OIAPOQUE	AP	20.509	27.270	0,658	0,566	0,668	0,694	100	84,11	69,30
6. TABATINGA	AM	52.272	65.844	0,616	0,554	0,631	0,661	61,22	59,40	71,94
7. GUAJARÁ-MIRIM	RO	41.656	46.174	0,657	0,602	0,609	0,558	16,40	68,86	73,33
8. PACARAIMA	RR	10.433	17.401	0,650	-	0,712	0,742	81,80	100	100
9. BONFIM	RR	10.943	12.409	0,626	0,593	0,503	0,732	100	100	100
**MIDWEST REGION**										
10. BELA VISTA	MS	23.181	24.629	0,698	0,679	0,681	0,616	72,76	85,46	100
11. CORUMBÁ	MS	103.703	111.435	0,700	0,611	0,625	0,558	58,02	59,88	82,07
12. MUNDO NOVO	MS	17.043	18.366	0,686	0,575	0,538	0,514	73,82	60,73	100
13. PARANHOS	MS	12.350	14.228	0,588	0,650	0,519	0,655	96,21	83,81	75,69
14. PONTA PORÃ	MS	77.872	92.526	0,701	0,623	0,623	0,604	40,40	53,16	62,61
15. CORONEL SAPUCAIA	MS	14.064	15.253	0,589	0,512	0,545	0,559	75,56	73,59	69,39
16. PONTO MURTINHO	MS	15.372	17.131	0,666	0,567	0,590	0,623	50,40	44,89	82,70
**SOUTH REGION**										
17. BARRACÃO	PR	9.735	10.275	0,706	0,588	0,592	0,587	100	100	100
18. FOZ DO IGUAÇU	PR	256.088	258.532	0,751	0,577	0,583	0,545	35,72	49,85	45,75
19. SANTO ANTÔNIO DO SUDOESTE	PR	18.893	20.166	0,671	0,580	0,557	0,524	37,94	91,30	100
20. GUAÍRA	PR	30.704	33.119	0,724	0,579	0,601	0,575	12,47	44,95	73,66
21. DIONÍSIO CERQUEIRA	SC	14.811	15.498	0,706	0,635	0,616	0,529	100	100	100
22. ACEGUÁ	RS	4.394	4.901	0,687	-	-	0,534	82,38	78,52	73,37
23. BARRA DO QUARAÍ	RS	4.012	4.215	0,662	-	0,605	0,504	-	100	100
24. CHUÍ	RS	5.917	6.704	0,706	-	0,533	0,495	48,15	0	53,80
25. ITAQUI	RS	38.159	37.620	0,713	0,615	0,591	0,523	24,16	27,12	44,18
26. JAGUARÃO	RS	27.931	26.680	0,707	0,569	0,553	0,500	-	24,70	73,33
27. PORTO XAVIER	RS	10.558	10.246	0,723	0,678	0,639	0,479	90,96	100	100
28. QUARAÍ	RS	23.021	22.687	0,704	0,617	0,567	0,518	-	44,96	87,88

**29. SANTANA DO LIVRAMENTO**	RS	82.464	77.027	0,727	0,616	0,608	0,554	-	-	45,93
**30. SÃO BORJA**	RS	61.671	60.282	0,736	0,644	0,601	0,547	66,16	78,32	45,93
**31. URUGUAIANA**	RS	125.435	126.970	0,744	0,584	0,604	0,578	5,6	5,50	55,85
**32. PORTO MAUÁ**	RS	2.542	2.374	0,698	-	0,389	0,421	100	100	100

**Subtitles:**	HDI-M:<br>High development<br>Medium development (0,600 – 0,699)<br>Low development (0,500 – 0,599)	GINI INDEX:<br>Little Unequal (< out of 0,400)<br>Unequal (0,550 – 0,401)<br>Very Unequal (> out of 0,550)	ESF COVERAGE (%):<br>HIGH COVERAGE (75-100%)<br>MEDIUM COVERAGE (50–74,9%)<br>LOW COVERAGE (0 – 49,9%).

.

Table **S3**. Average infant mortality rates by place of occurrence of infant death and place of maternal residence in the Twin Cities, Brasil, 2022.

Cities	1996-1997		1998- 1999		2000 -2001		2002 -2003		2004-2005		2006-2007		2008- 2009		2010-2011		2012-2013		2014-2015		2016-2017		2018-2019	
	O	R	O	R	O	R	O.	R	O	R	O	R	O	R	O	R	O	R	O	R	O	R	O	R
Região Norte																								
Assis Brasil - AC	26,83	24,93	-	-	-	-	-	-	10,47	17,77	-	-	-	-	-	-	9,22	13,83	-	-	-	-		-
Brasiléia – AC	51,17	29,76	-	-	10,16	8,67	14,04	15,57	*	*	5,05	13,55	6,97	18,59	8,21	13,81	8,60	23,11	12,45	20,50	7,62	34,70	6,01	12,13
Epitaciolândia - AC	-	-	733,3	583,3	550,0	356,1	350,0	38,7	155,6	284,5	154,8	23,3	176,9	29,5	-	-	-	-	-	-	-	-		14,9
Santa Rosa do Purus- AC	-	-	-	-	-	-	-	-	-	-	71,66	78,29	39,48	58,94	20,29	38,91	33,10	48,14	21,45	63,69	27,37	39,54	14,07	40,56
Oiapoque – AP	-	-	-	-	8,98	14,09	6,23	17,15	12,23	28,91	12,13	20,15	5,46	23,29	13,45	17,89	21,03	30,40	15,34	19,53	8,98	25,17	12,79	15,49
Tabatinga - AM	55,44	57,59	23,12	21,35	37,74	39,22	37,15	34,30	22,90	23,08	25,60	21,59	31,46	28,39	26,16	20,36	23,86	24,93	23,33	20,04	23,49	22,93	22,85	21,06
Guajará-mirim - RO	7,64	10,52	8,47	11,30	9,40	10,28	9,91	13,74	7,82	9,80	15,23	29,61	11,40	20,06	9,36	15,35	4,33	12,11	8,46	19,92	7,28	19,74	5,63	17,00
Pacaraima - RR	-	-	16,81	26,45	-	-	-	-	-	-	10,81	26,60	22,87	33,15	12,81	23,14	-	-	7,15	15,67	24,92	29,39	24,32	16,19
Bonfim – RR	-	-	22,09	34,47	25,67	31,73	-	-	-	-	23,19	29,59	-	-	10,94	17,69	-	-	-	-	-	-		
Região Centro-Oeste																								
Cáceres - MT	21,67	18,93	26,65	20,84	27,71	27,65	17,64	20,91	25,86	19,02	18,32	16,78	21,93	19,07	18,58	16,28	27,08	17,36	26,09	14,19	22,75	16,59	19,32	14,76
Bela Vista -MS	26,35	29,67	27,61	27,34	22,91	22,52	28,97	38,08	22,52	33,92	23,19	27,16	16,70	28,95	9,02	17,25	7,35	15,95	6,72	8,91	8,43	13,86	9,74	15,29
Corumbá- MS	52,89	50,04	41,27	41,83	33,58	34,02	32,38	32,14	24,93	24,10	17,10	18,78	20,09	22,02	19,01	18,42	16,36	16,86	18,28	21,67	14,53	18,44	11,64	13,88
Mundo Novo - MS	25,81	22,96	16,42	25,02	21,18	28,63	18,86	26,06	12,80	17,05	19,17	29,35	30,42	26,06	14,51	21,00	-	-	13,65	18,79	15,22	21,39	7,59	12,71
Paranhos - MS	80,68	60,75	30,25	38,20	52,14	64,15	42,30	58,21	37,84	52,87	29,61	43,53	17,59	36,83	20,60	31,26	12,39	25,32	16,35	29,32	-	-	21,32	31,19
Ponta Porã- MS	29,82	36,02	23,94	29,27	23,23	28,44	21,59	27,89	16,52	21,33	13,36	18,59	11,87	19,47	8,53	16,91	5,53	14,01	3,73	13,19	4,91	15,78	3,15	16,64
Coronel Sapucaia - MS	38,00	42,59	40,16	56,26	13,65	32,71	18,71	54,64	25,68	53,82	29,33	45,29	28,47	32,26	9,35	23,14	11,50	18,97	19,50	26,69	12,11	17,90	9,05	15,63
Ponto Murtinho - MS	52,80	66,38	30,56	36,71	38,09	43,90	23,19	37,52	27,10	28,32	14,92	21,89	18,86	19,53	11,11	14,95	16,99	22,44	10,74	16,98	7,38	10,80	7.99	13,18
Região Sul																								
Barracão – PR	26,98	24,43	26,55	28,37	-	-	-	-	30,83	33,72	-	-	-	-	-	-	-	-	-	-	-	-	-	28,77
Foz do Iguaçu- PR	8,27	8,06	-	-	21,36	19,68	15,92	14,49	13,88	13,60	15,82	14,88	14,04	12,66	12,32	10,91	14,05	11,96	16,71	14,52	14,15	11,73	13,41	10,88
Santo Antônio do Sudoeste- PR	4,90	11,40	5,52	6,50	-	-	7,80	10,98	-	-	4,50	13,49	15,35	19,91	-	-	-	-	-	-	-	-	-	-
Guaíra- PR	20,29	24,21	28,38	34,38	15,84	18,13	14,08	18,22	8,19	11,06	10,24	14,82	12,72	17,68	8,83	14,47	7,21	16,08	6,67	16,00	6,47	16,65	-	10,41
Dionísio Cerqueira- SC	23,75	23,40	15,81	25,59	7,57	10,78	19,87	21,13	22,25	29,80	11,25	16,03	7,38	11,78	4,32	6,45	4,32	12,40	5,93	15,75	-	-	-	-
*Aceguá – RS***	-	-	-	-	-	-	-	-	-	-	-	-	-	-	-	-	-	-	-	-	-	-	-	-
*Barra do Quaraí- RS***	-	-	-	-	-	-	-	-	-	-	-	-	-	-	-	-	-	-	-	-	-	-		

Cities	*1996-1997*		*1998- 1999*		*2000 -2001*		*2002 -2003*		*2004-2005*		*2006-2007*		*2008- 2009*		*2010-2011*		*2012-2013*		*2014-2015*		*2016-2017*		*2018-2019*	
	O	*R*	*O*	*R*	*O*	*R*	*O.*	*R*	*O*	*R*	*O*	*R*	*O*	*R*	*O*	*R*	*O*	*R*	*O*	*R*	*O*	*R*	*O*	*R*
*Chuí- RS***	-	-	-	-	-	-	-	-	-	-	-	-	-	-	-	-	-	-	-	19,98	-	-		
Itaqui – RS	13,87	17,83	16,82	18,30	11,56	16,21	9,68	13,74	8,75	13,44	7,70	13,96	2,65	4,31	5,20	13,20	3,78	12,56	11,78	14,45	6,58	13,56	7,82	9,82
Jaguarão – RS	15,39	21,13	10,39	25,76	6,36	17,07	9,91	18,13	-	-	5,74	19,31	3,02	14,40	-	-	3,53	7,04	-	-	7,71	18,80	3,67	13,34
Porto Xavier – RS	12,88	13,37	16,18	19,88	12,74	14,82	23,37	32,39	13,47	10,33	17,15	15,10	-	-	-	-	11,70	15,10	-	-	-	-	-	-
Quaraí – RS	31,25	31,88	25,01	25,49	23,43	31,67	22,93	26,92	7,50	10,26	20,64	27,41	-	-	9,38	12,68	6,10	10,64	-	-	10,62	21,67	-	-
Santana do Livramento - RS	22,04	22,92	23,39	24,01	16,40	15,53	21,44	25,57	18,58	24,36	13,20	17,18	8,26	11,11	8,18	17,52	8,47	13,35	3,86	9,10	8,38	14,06	7,74	14,87
São Borja – RS	11,47	11,12	15,15	16,33	9,79	10,33	14,02	15,38	9,80	15,38	12,53	17,22	8,52	11,34	13,82	13,35	12,19	16,87	11,75	15,11	7,93	13,52	5,78	9,47
Uruguaiana – RS	31,00	30,25	29,95	30,58	25,27	26,49	24,66	26,96	23,01	22,77	18,06	18,29	18,76	18,48	21,64	20,00	17,49	17,48	15,86	15,34	87,89	13,72	12,16	12,21
Porto Mauá- RS	-	-	-	-	-	-	-	-	-	-	-	-	-	-	-	-	-	-	-	-	-	-	7,82	9,82
Cidades gêmeas	29,11	29,18	53,35	51,60	43,35	38,92	34,22	26,87	24,21	35,46	23,15	24,42	23,01	22,55	12,59	18,12	11,78	18,16	12,49	19,76	15,60	19,67	11,48	16,54

No official information available through the /DATASUS system for certain years or not enough data to perform the averages.

O - TMI by Occurrence

R - TMI per Residence

IMR Classification Signaling		
(blue)	Low	less than 20 deaths
(yellow)	Medium	20-49 deaths
(red)	High	50 deaths or more
